# Prevalence of intestinal parasitic infection and its associated factors among primary school students in Ethiopia: A systematic review and meta-analysis

**DOI:** 10.1371/journal.pntd.0009379

**Published:** 2021-04-27

**Authors:** Moges Agazhe Assemie, Daniel Shitu Getahun, Yidersail Hune, Pammla Petrucka, Ayele Mamo Abebe, Animut Takele Telayneh, Mekdes Marew Ambaw, Daniel Bekele Ketema, Temesgen Getaneh, Belayneh Mengist, Muluneh Alene, Samuel Derbie Habtegiorgis

**Affiliations:** 1 Biostatstics Unit, Department of Public Health, College of Health Science, Debre Markos University, Debre Markos, Ethiopia; 2 Reproductive Health Unit, Department of Public Health, College of Health Science, Debre Markos University, Debre Markos, Ethiopia; 3 Department of Human Nutrition, College of Health Science, Debre Markos University, Debre Markos, Ethiopia; 4 College of Nursing, University of Saskatchewan, Saskatoon, Canada; 5 School of Life Sciences and Bioengineering, Nelson Mandela African Institute of Science and Technology, Arusha, Tanzania; 6 Department of Pediatrics and Child Health, College of Health Science, Debre Berhan University, Debre Berhan, Ethiopia; 7 Epidemiology Unit, Department of Public Health, College of Health Science, Debre Markos University, Debre Markos, Ethiopia; 8 Department of Agricultural Economics, College of Agriculture and Resource Management, Dilla University, Dilla, Ethiopia; 9 Department of Midwifery, College of Health Science, Debre Markos University, Debre Markos, Ethiopia; Ben-Gurion University of the Negev, ISRAEL

## Abstract

**Introduction:**

Intestinal parasitic infection are a major public health concern affecting both children and adolescents in Ethiopia. The aim of this systematic review and meta-analysis was to determine pooled prevalence and associated factors of intestinal parasitic infection in this target group.

**Method:**

We systematically retrieved available articles on the prevalence of intestinal parasitic infection following database searches using PubMed, Scopus, Cochrane Library, and Science Direct between March 1 and May 27, 2020. Two authors independently extracted all relevant data using a standardized Microsoft Excel data extraction form. Heterogeneity among included studies was assessed with the Higgins I^2^ tests. The pooled estimates and associated factors were assessed with a random-effects model using Stata/se Version 14.

**Result:**

We retrieved 30 eligible articles with a pooled sample size of 14,445 primary school children with response rate of 97.8%. Entamoeba spp (16.11%), Ascaris lumbricoides (13.98%), hookworm (12.51%) and Giardia lamblia (9.98%) are the top causes of intestinal parasitic infection among primary school children in Ethiopia. The pooled prevalence for at least one intestinal parasitic infection was 46.09 (95% CI: 38.50, 53.68). Heterogeneity was assessed by doing subgroup analysis by study province/region. Thus, the highest prevalence of 66.6% (95% CI: 55.5, 77.7) occurred in Tigray region, which was followed by Southern Nations, Nationalities, and Peoples’ Region at 50.8% (95% CI: 33.1, 68.5). No latrine availability (OR = 4.39: 2.50,7.73), no fingernail hygiene (OR = 2.37: 1.67, 3.35), open defecation (OR = 1.67:1.64,4.36), no formal maternal education (OR = 2.02: 1.18,3.47), rural residence (OR = 1.88: 1.46, 2.41), no habit of wearing shoes (OR = 2.66: 1.79, 3.96), non-pipe source of drinking water (OR = 1.99: 1.42,2.76), no regular hand washing practices (OR = 3.45:1.85,6.47), and no habit of washing fruits and vegetables (OR = 1.59:1.01,2.49) were associated with parasitic infection.

**Conclusions:**

The prevalence of intestinal parasitic infection was high (46%). Attention should be given to promoting personal hygiene, latrine utilization, wearing shoes, avoiding eating raw food, creating awareness for those mothers who lack formal education. Moreover, future research ideally will expand on the topic by conducting research in regions which have no prior research.

## Author summary

Intestinal parasitic infections are distributed virtually with high prevalence rates in many regions, especially in low and middle income countries which intimately linked to age group, poverty and inadequate access to water, sanitation and hygiene. It is now a day’s one of the neglected tropical disease. We had searched different individual studies on intestinal parasitic infections to generate the pooled effect and recommend for the concerned body. We head included 30 different articles with a sample size of 14,445 and we have found a pooled prevalence of 46.09%. This finding indicates the prevalence of parasitic infection was almost near to half. Therefore every concerned body should give emphasize to prevent and control parasitic infection infestation.

## Introduction

Intestinal parasitosis refers to a group of diseases caused by one or more species of protozoa, cestodes, trematodes or nematodes distributed with high prevalence throughout the world [1]. Amoebiasis, ascariasis, hookworm infection, and Trichuris are among the most common parasitic infections [2, 3]. More than 550 million school children live in areas where intestinal parasitic infections (IPI) are endemic with 450 million of the illnesses occurring in sub-Saharan Africa of which Ethiopia is second only to Nigeria in prevalence [2, 4–7] Parasitic infections are preventable neglected tropical infections [8], yet it is common among primary school children in communities where socioeconomic status and hygienic condition are poor [2, 9]. The effects of IPI are not limited to morbidity and mortality, but also extend to nutritional problems (i.e., stunted growth, low vitamin A, iron deficiency anemia, loss of weight, chronic blood loss), psychological and social wellbeing [10, 11], as well as compromised mental development (i.e, impaired growth, decreased school attendance, cognitive impairment, decreased educational achievement and adult productivity) [12, 13]. Additionally, IPI increases susceptibility to diarrhea, infections like HIV, and other infectious diseases [14].

Although there were many studies investigating prevalence of IPI among primary school children, the reported findings were inconsistent and highly variable (ranging from 10.9% to 81.0%) [15–17]. Additionally, no systematic review and meta-analysis has been done to enhance the quality and consistency of the evidence. The aim of this systematic review and meta-analysis is to determine the pooled prevalence of IPI and associated factors among primary school children in Ethiopia using available evidence. This study will inform policy makers and program planners in their efforts to design a school-centric survey and implement efficient interventions to decrease the burden and impacts of IPI among primary school children.

## Methods

### Search strategy

Two authors (SDH and DS) independently searched all articles that reported the prevalence of IPIs from Medline/PubMed, Google Scholar, Science Direct, HINARI, and Cochrane Library (S1 **Table**). Our search was extended by hand searches for grey literature and retrieving reference lists of eligible articles. The literature search occurred between March 1 and May 27, 2020 using common Boolean words: prevalence AND intestinal parastic infection OR parastic infection AND associated factors AND primary school children AND Ethiopia. We used the Preferred Reporting Items for Systematic Reviews and Meta-Analyses (PRISMA) guidelines to present this systematic review and meta-analysis.

### Eligibility criteria

#### Inclusion criteria

Criteria were established for eligibility before beginning the search. All observational published and unpublished articles reporting the prevalence of at least one IPI among primary school children in Ethiopia were included.

#### Exclusion criteria

Case reports and reviews were excluded. Moreover, articles which were not fully accessible, after at least two email contacts with the primary authors, were excluded because of the inability to assess the quality of articles in the absence of full text.

### Data extraction

Independently two authors (YH and MMA) extracted all data using a uniform extraction format prepared in Microsoft Excel. The data extraction format included first author, publication year, study area, sample size, age of participant, response rate, and prevalence of IPI. Any disagreements among authors during extraction were resolved by discussion and mutual agreement with the help of a third author (ATT).

### Outcome measurements

This systematic review and meta-analysis had two objectives. The first objective was to determine the reported prevalence of IPI which is ascertained using the formal-ether concentration technique following standard protocol. The prevalence was calculated by dividing the number of infected children with at least one IPI by the total number of participants who have been included in the study (sample size) multiplied by 100. The second objective of the study was to identify the factors associated with the IPI. The odds ratios of associated factors were calculated using the two by two tables with binary outcomes sex (male/female), residence (urban/rural), age of children (≤10years/>10years), family size (<5/ ≥5), habit of eating raw meat (always/sometimes), waste disposal (open/not open), defecation (latrine/open field), latrine availability (yes/no), maternal education (literate/illiterate), habit of eating raw vegetables (always/sometimes), habit of washing fruit and vegetables (yes/no), habit of swimming (yes/no), habit of washing hands after toilet (yes/no), hand washing before meal (yes/no), source of drinking water (pipe/not pipe), habit of wearing shoes (yes/no), and hygiene of fingernails (yes/no).

### Quality assessment

To assess the quality of studies, we used the Newcastle-Ottawa Scale quality assessment tool adapted for cross-sectional studies. The tool has three main sections. The first section has the potential for five stars and assesses the methodological quality of each study. The second section of the tool evaluates the comparability of the studies and has potentially two stars. The last part of the tool has a potential for three stars to measure the quality of the original articles with respect to their statistical analysis. Articles attaining the score of 5 and above out of 10 were considered as high quality and included for analysis [18] (**S2 Table**).

### Statistical data analysis

The analysis was done by STATA/se Version 14.0 statistical software. We presented results with tables and forest plots. Heterogeneity among studies was assessed by calculating p-values for Higgins I^2^- statistics. The pooled prevalence of IPI was estimated with a random effect model by generating the pooled 95% confidence interval using the Der Simonian and Laird’s methods. Moreover, univariate meta-regression model was conducted by taking the publication year and the sample size of the studies to identify the possible sources of heterogeneity. Publication bias was also assessed using funnel plot and Egger’s regression intercept tests at 5% significant level. In addition, to minimize the random variations between the point estimates of the primary studies, subgroup analysis was done based on region of studies, publication year, and/or sample size.

## Results

### Description of identified studies

**Fig 1:**
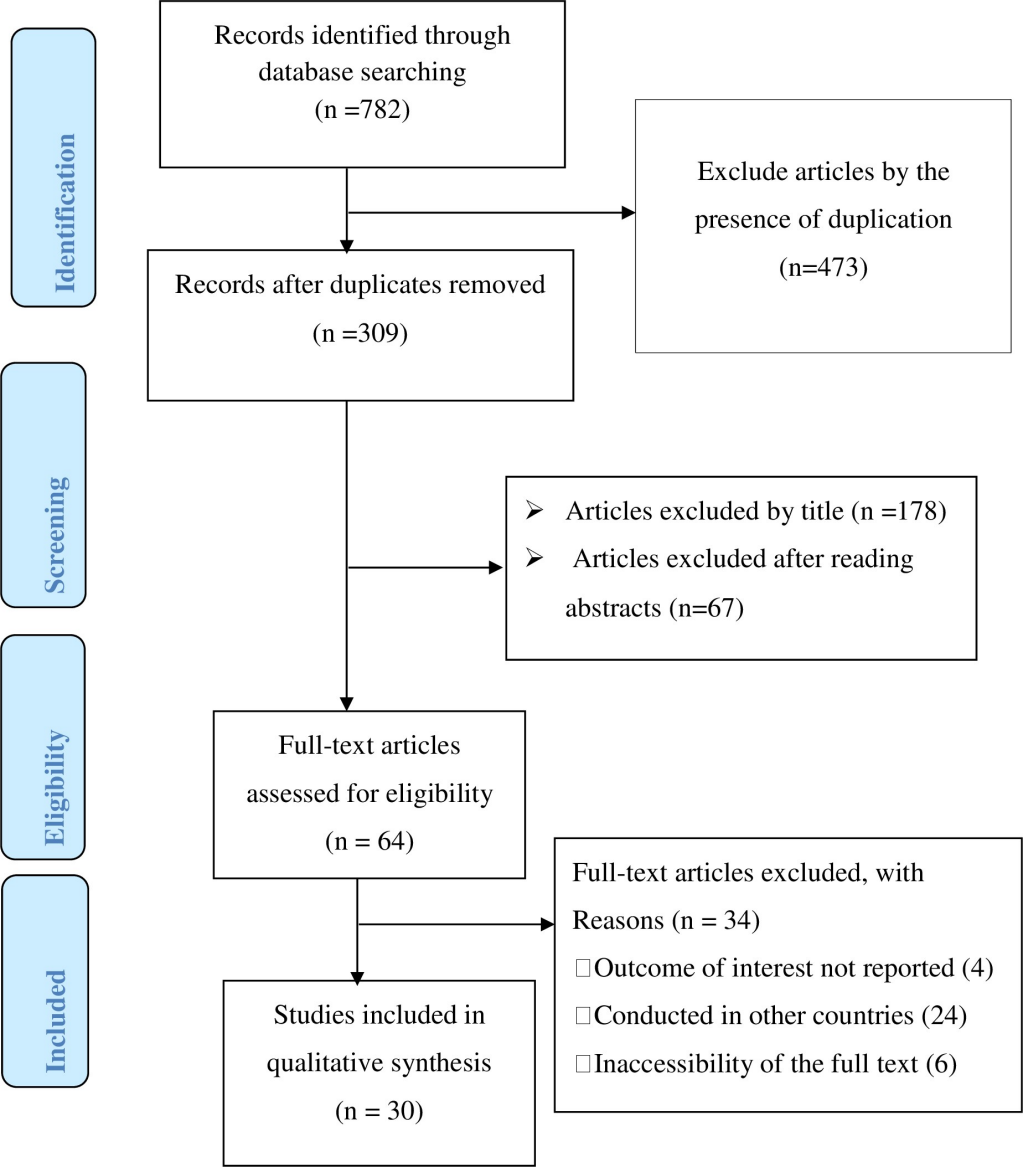
Describe the flow chart of selecting articles eligible for systematic review and meta-analysis.

The review of electronic databases and reference lists from relevant studies yielded 782 potential articles. After excluding 473 articles because of duplication, the remaining articles were screened based on the pre-set eligibility criteria. Then, after reviewing the titles and abstracts of 309 articles, 245 articles were excluded. From the remaining 64 articles, only 30 articles were found eligible and included in the final meta-analysis after reading the full texts and assessing for meeting the inclusion criteria (Fig 1).

### Characteristics of original articles

The characteristics of 30 articles included in this systematic review and meta-analysis was summarized in **Table 1**. All studies included were cross-sectional study design. The sample size of the included studies range from 172 (in Addis Ababa) [19] to 2,372 (in Bahir Dar and Mecha District) [20]. A total of 14,455 primary school children with a response rate of 97.8% participated to estimate the pooled prevalence of IPI. Of the total nine regions and two city administrations in Ethiopia, five regions and one city administration were include; specifically, Addis Ababa [19], Amhara [15, 17, 20–32], Oromia [16, 33–35], Tigray [36, 37], SNNPR [38–43] and Benishangule Gumuz [44]. The highest prevalence (81.0%) was reported in a study conducted in Chencha town, Gamo Gofa Zone (SNNPR) [39] while the lowest prevalence (10.9%) was noted from a study in Gondar town, North West Ethiopia (Amhara) [25].

**Table 1:** Characteristics of 30 studies reporting the prevalence of intestinal parasite infection.

Authors	Pub/year	Area	Sample	Response rate (%)	Prevalence (%)
Sitotaw et al[17]	2019	Amhara (Jawi)	422	96.2	57.9
Nguyen et al[21]	2012	Amhara (Angolela)	664	100	37.2
Feleke[20]	2016	Amhara (Mecha & BDR)	2372	94.54	61.7
Alelign et al[23]	2015	Amhara (Durbete)	403	95.29	54.95
Amare et al[24]	2013	Amhara (Gondar)	405	100	22.7
Worku N et al[25]	2009	Amhara (Gondar)	322	100	10.9
Hailegebriel[22]	2017	Amhara (Bahir Dar)	390	92.1	65.5
Gashaw et al[26]	2015	Amhara (Enfranz)	550	100	66.4
Alemu et al [15]	2019	Amhara (Lay Gayint)	273	100	30.8
Dessie et al[28]	2019	Amhara (Glomekeda)	422	100	29.9
Gelaw et al[29]	2013	Amhara	326	93.25	34.2
Melaku et al[45]	2019	Amhara (Bahir dar)	211	100	60.2
Asemahagn[31]	2014	Amhara (Motta)	358	98.3	68.4
Abdi et al [32]	2017	Amhara (Zegie)	422	96.68	69.1
Sisay et al[33]	2019	Oromia (Zwai)	384	100	22.6
Tefera et al[33]	2017	Oromia (Babile)	632	100	14.4
Begna[34]	2016	Oromia (Bale zone)	492	100	26.6
Reji P et al[46]	2011	Oromia (Adama)	358	100	35.5
Geleta et al[35]	2018	Oromia (Arsi Negele)	295	100	39.6
Mengist et al[19]	2015	Addis Ababa	172	97.8	37.8
Gebretsadik[44]	2017	BG(Asossa)	404	97.77	35.44
Mahmud et al[36]	2013	Tigray(Mekele)	600	97.2	72
Kidane et al[37]	2014	Tigray (Wukro)	384	100	60.7
Zerdo et al[47]	2017	SNNP (Gamo Goffa)	406	100	36.8
Wolde et al[43]	2015	SNNP (Sidam zone)	450	98.8	64.3
Birmeka., et al[42]	2017	SNNP (Gurage zone)	641	100	40.2
Alemu et al[41]	2018	SNNP (Arbaminch)	405	96.54	46.5
Alemayehu et al [38]	2017	SNNP (Wolaita zone)	515	97.67	72.2
Alemu et al[40]	2019	SNNP (Birbir town)	355	98.87	27.1
Abossie et al[39]	2014	SNNP (Chencha town)	422	94.79	81

### Meta-analysis

#### Species of intestinal parasitic infection

The highest prevalent IPI species among primary school students were Entamoeba spp. with a prevalence of 16.11% (95% CI: 10.79, 21.43) followed by Ascaris lumbricoides 13.98% (95% CI: 9.30, 18.67) whereas the least prevalent parasite reported was Strongloids stecolaris 1.56% (95% CI: 0.88, 2.24) (Table 2).

**Table 2:** Subgroup analysis of intestinal parasite infection by parasite species.

Parasite species	Study Number	Participants	Prevalence with 95% CI
Entamoeba spp.	15	6159	16.11(10.79, 21.43)
Ascaris lumbricoides	16	6550	13.98(9.30, 18.67)
Hookworm	16	6720	12.51(8.73, 16.28)
Giardia lamblia	13	5283	9.98(6.91, 13.05)
Hymenolepis nana	14	5770	7.82(5.24, 10.40)
Schistosoma mansoni	5	2189	5.37(1.86, 8.89)
Trichuris trichiura	14	5802	3.51(2.31, 4.70)
Enterobius vermicularis	10	4151	2.34(1.16, 3.52)
Taenia spp.	8	3450	1.60(0.77, 2.44)
Strongyloides stercoralis	8	3296	1.56(0.88, 2.24)

**Fig 2:**
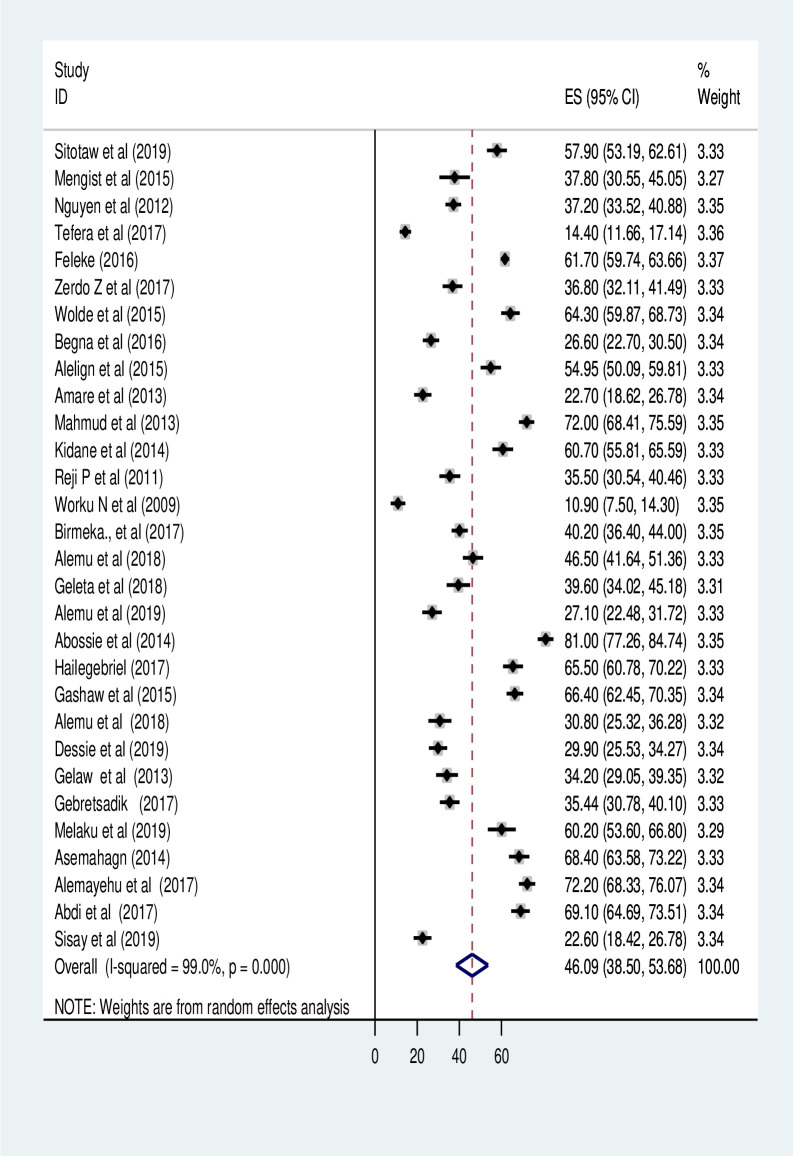
Forest plot of the pooled prevalence of intestinal parasitic infection.

The pooled prevalence of at least one IPI among primary school children was found to be 46.09% (95% CI: 38.50, 53.68) (I^2^ = 99.0%, p<0.001) (**Fig 2**).

### Subgroup analysis

To investigate the source of heterogeneity between studies, we performed subgroup analysis by region where the study conducted. The highest prevalence was observed in Tigray region with a prevalence of 66.6% (95% CI: 55.5, 77.7), followed by SNNPR at 50.8% (95% CI: 33.1, 68.5), and the lowest was noted in Oromia region at 27.58% (95% CI: 18.6, 36.6). In addition, we conducted subgroup analysis by sample size below and above mean. The prevalence of IPI was relatively similar to the overall pooled prevalence across the category above and below the mean sample size 45.99% (95% CI: 36.68, 61.48). Finally, subgroup analysis was done for publication year, with the finding that before 2017 a higher prevalence was seen compared to studies published after 2017(inclusive) (Table 3).

**Table 3:** Subgroup analysis of intestinal parasite infection by Region.

Variables	Characteristics	No. of Studies	Participants	Prevalence(95% CI)
Region	Amhara	14	7540	47.84 (37.14, 58.52)
	SNNP	7	3194	50.84 (33.14, 68.53)
	Oromia	5	2161	27.58 (18.62, 36.55)
	Tigray	2	984	66.60 (55.54, 77.66)
	Addis Ababa	1	172	37.80 (30.55, 45.05)
	Benishangul Gumuz	1	404	35.44 (30.78, 40.10)
Sample size	Below mean(482)	22	7989	44.81 (36.69, 52.93)
	Above mean(482)	8	6466	45.99 (36.68, 61.48)
Publication year	Before 2017	15	8278	48.97 (37.99, 59.95)
	After 2017(inclusive)	15	6177	43.19 (33.07, 53.31)

**Fig 3:**
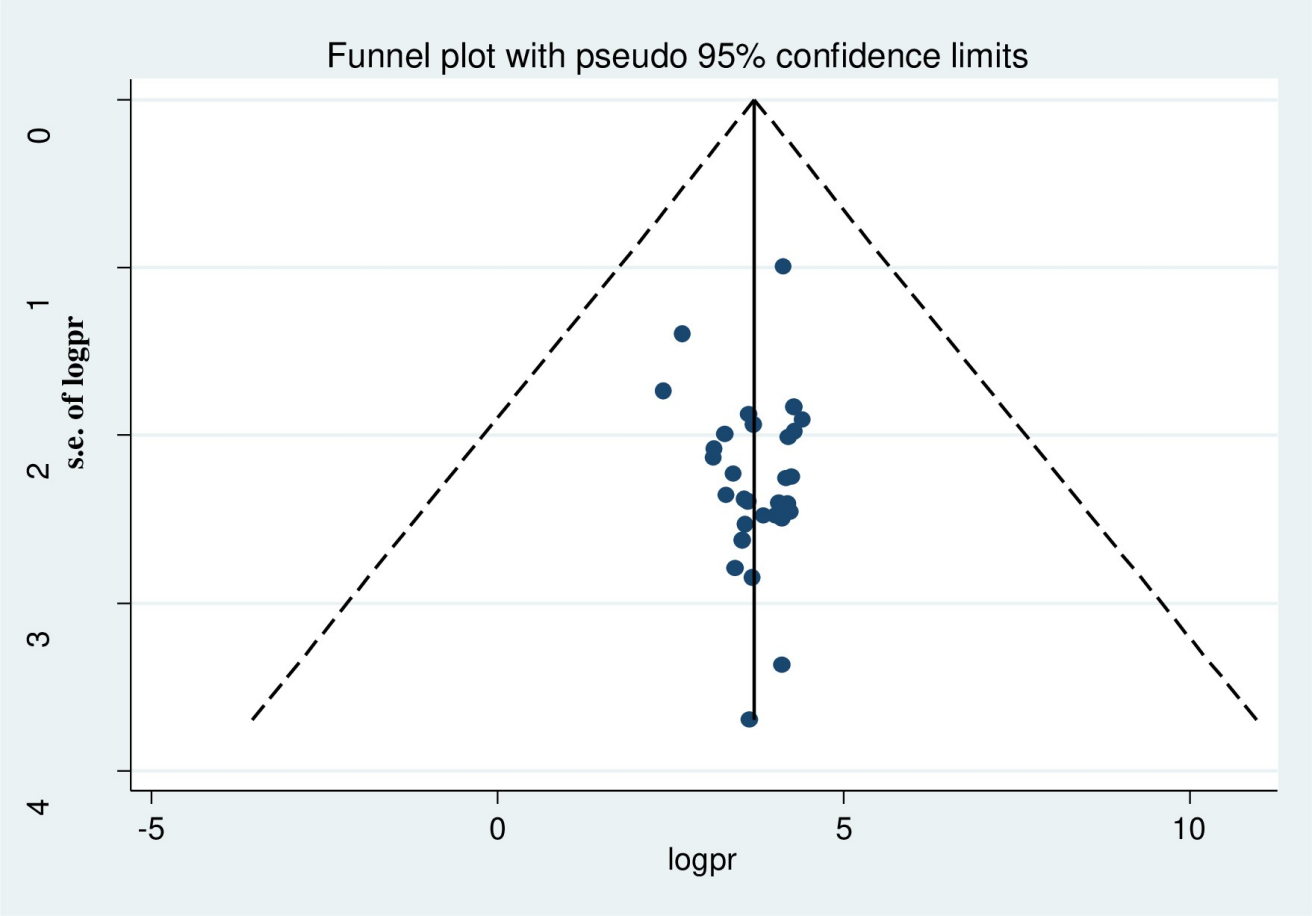
Funnel plot with 95% confidence limits of the pooled prevalence of intestinal parasitic infection.

To determine the extent of publication bias, we performed the funnel plot for symmetry by visual inspection, and it appeared quite symmetrical (Fig 3) indicating the absence of publication bias. To confirm the status of publication bias we also performed Egger’s objectivity test, which also did not show presence of publication bias (p> 0.758).

### Factors associated with intestinal parasite infection

In this systematic review and meta-analysis; hygiene of fingernail was reported from seven articles, place of defecation from six articles, latrine availability from seven articles, maternal education from eight articles, residence from four articles [4], habit of shoe wearing from elven articles source of drinking water from nine articleshands washing habit from seven articles, and habit of washing fruit and vegetables from three articles were associated with IPI. However, family size reported from five articles, sex from fifteen articles, age of children from elven articles, habit of eating raw meat from four articles, habit of swimming from seven articles, habit of eating raw vegetables from five articles, hand washing before meals from three articles and waste disposal sites from three articleswere not associated with IPI (Table 4).

**Table 4:** Factors associated with intestinal parasitic infections.

Variables	Number of articles	Pooled odds ratio (95% CI)	I-squared (%)	I^2^ p-value
Age of students below 10 years	11	1.28(0.99, 1.65)	73.3	<0.001
Female	15	1.04(0.99, 1.20)	38.6	0.063
No latrine availability	7	4.39(2.50, 7.73)*	64.8	0.009
Had no fingernail hygiene	7	2.37(1.67, 3.35)*	80.8	<0.001
open defecation	6	1.67(1.64, 4.36)*	85.3	<0.001
No formal maternal education	8	2.02(1.18, 3.47)*	89.3	<0.001
Rural residence	4	1.88(1.46, 2.41)*	0	0.667
No habit of shoes wearing	11	2.66(1.79, 3.96)*	87.7	<0.001
Non-pipe drinking water	9	1.99(1.42, 2.76)*	60.8	0.009
No hands washing habit	7	3.45(1.85, 6.47)*	87.2	<0.001
No habit of washing fruit	3	1.68(1.23, 2.30)*	47.2	0.151
Family size above 5	5	1.60(0.70, 3.80)	78.8	0.001
Habit of eating raw meat	4	0.97(0.37, 2.51)	92.9	<0.001
Habit of swimming	7	1.19(0.61, 2.31)	93.2	<0.001
Habit of eating raw vegetables	5	1.05(0.31, 3.53)	98.1	<0.001
No hand washing before meal	3	3.31(0.70, 15.53)	92.7	<0.001
Open waste disposal site	3	1.08(0.16, 7.29)	98.1	<0.001

*statistically significant variable with its CI

The association between shoes wearing habit and intestinal parasitic infection was determined from elven articles with sample size of 6,229 [16, 17, 22, 28, 31, 32, 35, 40, 41, 44, 48]. Thus, we found those individual who do not regularly wear shoes was 2.7 times more likely to develop IPI (OR: 2.66, 95% CI: 1.79, 3.97) as compared to those who habitually wear shoes (Table 4).

The association between fingernail hygiene and intestinal parasitic infection was determined from seven studies with a sample size of 5,155[16, 17, 22, 28, 35, 48, 49]. The result of this analysis shows significant association between fingernail hygiene and IPI. The likelihood of parasitic infection is 2.4 times higher among students who had no fingernail hygiene as compared to their counterparts who have good fingernail hygiene (OR: 2.37, 95% CI: 1.67, 3.35) (Table 4).

The association between washing fruits and vegetables and IPI was determined from three papers with a sample size of 945 participants [32, 40, 41] which was found to be significant. Students not washing fruits and vegetables were 68% (OR: 1.68, 95% CI: 1.23, 2.30) more likely to be infected by intestinal parasites as compared to their counterparts (Table 4).

The association between maternal education and IPI was determined from eight articles with sample of 3,279 participants[22, 31, 35, 39, 40, 42, 44, 46]. The finding of meta-analysis indicates the significant association between maternal education and IPI. Those students who had an educated mother were two times less likely to be infected by intestinal parasite (OR: 2.02, 95% CI: 1.17, 3.48) as compared to uneducated one (Table 4).

The association between defecation habits and IPI was determined from six studies with 4,337 participants [17, 20, 28, 31, 32]. This meta-analysis indicated significant association between the defecation habits and IPI. Students who use open defecation were 2.7 times (OR: 2.67, 95% CI: 1.64, 4.37) higher to be infected by parasites as compared to those utilizing latrines (Table 4).

The association between hand washing habits and IPI was determined from six studies with a sample size of 4,337 [16, 17, 20, 28, 31, 32]. We had found significant association between hand washing and IPI. Students, who did not have regular hand washing habits, had more than triple the likelihood (OR: 3.45, 95%CI: 1.85, 6.47) of being infected by intestinal parasite as compared to those participating in regular hand washing (Table 4).

The association between residence and IPI was determined from four articles with 1,295 participants [17, 19, 31, 40]. Those who live in the rural area were 87% (OR: 1.87, 95% CI: 1.45, 2.41) more likely to develop IPI as compared to those living in urban areas (Table 4).

The association between IPI and source of drinking water were determined from nine articles [17, 31, 32, 35, 39–42, 50]. Non pipe source of drinking water is statistically associated with IPI. Thus, students who drank water from non-pipe sources were 99% (OR = 1.99, 95% CI: 1.99, 2.79) more likely to become infected with parasite as compared to pipe sources users (Table 4).

The association between IPI and latrine availability was determined from seven studies. We found latrine availability is statistically associated with IPI, with students who had no latrine being four times (OR = 4.39, 95% CI: 2.50, 7.73) more likely to be infected by intestinal parasites as compared to those with latrine availability (Table 4).

## Discussion

The findings display the prevalence of IPI among the primary school children in the country Ethiopia. Entamoeba spp (16.11%), Ascaris lumbricoides (13.98), Hookworm (12.51%) and Giardia lamblia (9.98%) are among the most prevalent parasitic infections among primary school children.

The overall pooled prevalence of IPI was 46.09%. This finding was in line with the studies conducted in Kenya (43%) [51], Nigeria (42.6%) [52], and Palestine (32.0–41.5%) [53]. On the other hand, studies that showed higher prevalence than our findings were done in West Africa (63.1%) [54], South Africa (68%) [55], Tanzania (63.91%) [56], Nigeria (67.4%) [57, 58], and Mkhanyakude District, Burkina Faso (84.7%) [59]. However, our results were higher than studies conducted in South Africa (37.5%) [60], Eastern Region of Nepal (31.5%) [61], Northwestern Iran (10.6%) [62], Tehran, Iran (18.4%) [63], and Yemen (31.8%) [64]. These variations could be due to socio-demographic characteristics, sample sizes, methodology used, and economic status.

Primary school children wearing shoes regularly were less likely to develop IPI than those not wearing shoes. This finding was supported by different studies in different areas [65–67], as a result of soil-transmitted helminthes infections (STHI) being a common source of IPI in humans. This trend could be explained by STHIs are transmitted by skin penetration that shoes are an effective way to block infection with STHI that penetrate the skin.

School children who did not maintain fingernail hygiene were more likely to be infected by IPI as supported by a study in Yemen [68]. This finding may arise because being rural community dwellers had lower awareness and more frequent contact with soil as well limited access to water and unable to maintain fingernail hygiene. Poor fingernail hygiene is also associated with poor socioeconomic status as there may be a lack of hygiene materials like water and soap. Students belonging to uneducated mothers at a higher risk of IPI in our study, which is a similar finding to studies in Turkey and El Beheira, Egypt [69, 70]. This finding could reflect that educated mothers had better knowledge about IPI prevention and potentially better economic status enabling them to embrace the entire prevention process with available materials like soap/ash, water, shoes and other necessities. Additionally, in the current study, rural residents were 87% more likely to be infected by intestinal parasites, which is similar to findings from studies in Zambia and Nepal [71, 72]. This situation may arise because rural community dwellers had lower awareness and more frequent contact with soil as well limited access to safe water and latrine. Moreover, there might be more likelihood of travel to lakes and ponds placing these people at greater risk.

Like other studies we were also found fruit and vegetable washing affected the prevalence of IPI [73], which might relate to the level of cleanliness and soil transmission risks. Open defecation and the availability latrines were other potential causes of IPI in our findings, which is similar to a study done in Zambia [71]. This finding could reflect that those communities who experience unclean environments due to open defecation are more likely to transmit parasites through soil, water, fomites, foods, or other means. Similar to other studies, hand washing habits, before meals, after toileting, and post-contact with dirty objects were significantly associated with IPI [65, 74].

## Limitations of the study

One of the limitations of this study was that it did not consider all the regions in Ethiopia due to lack of availability of articles.

## Conclusion

In this meta-analysis, the prevalence of intestinal parasitic infection in primary school children in Ethiopia was high (46%). Attention should be given to promoting personal hygiene, latrine utilization, wearing shoes, avoiding eating raw foods, and creating awareness for those mothers who lack formal education. Moreover, the researchers try to conduct research on regions which have no prior research.

## Supporting information

S1 Table Sample searches for the databases to assess the prevalence and associated factors of intestinal parasitic infection among primary school children in Ethiopia. (TIF)

S2 Table Assessing the quality of the 30 included studies on intestinal parasitic infection among primary school children in Ethiopia. (TIF)
