# Supplementary figures and images for: Prevalence of intestinal parasitic infection and its associated factors among primary school students in Ethiopia: A systematic review and meta-analysis
Source: PLoS Negl Trop Dis. 2021 Apr 27;15(4):e0009379. doi: 10.1371/journal.pntd.0009379 (PMC8104388; doi:10.1371/journal.pntd.0009379)

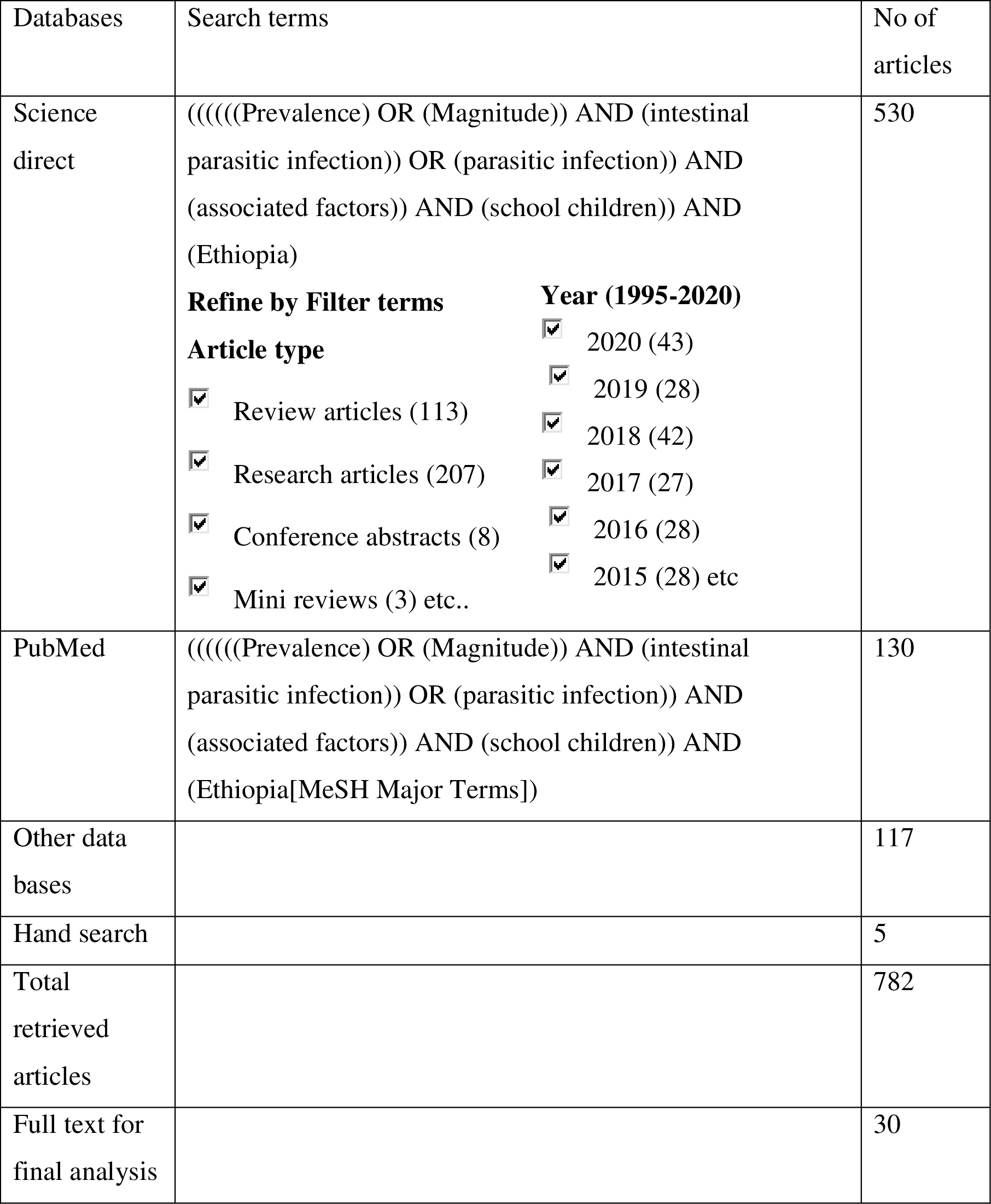

Supplement: S1 Table — (TIF) [file pntd.0009379.s001.tif]

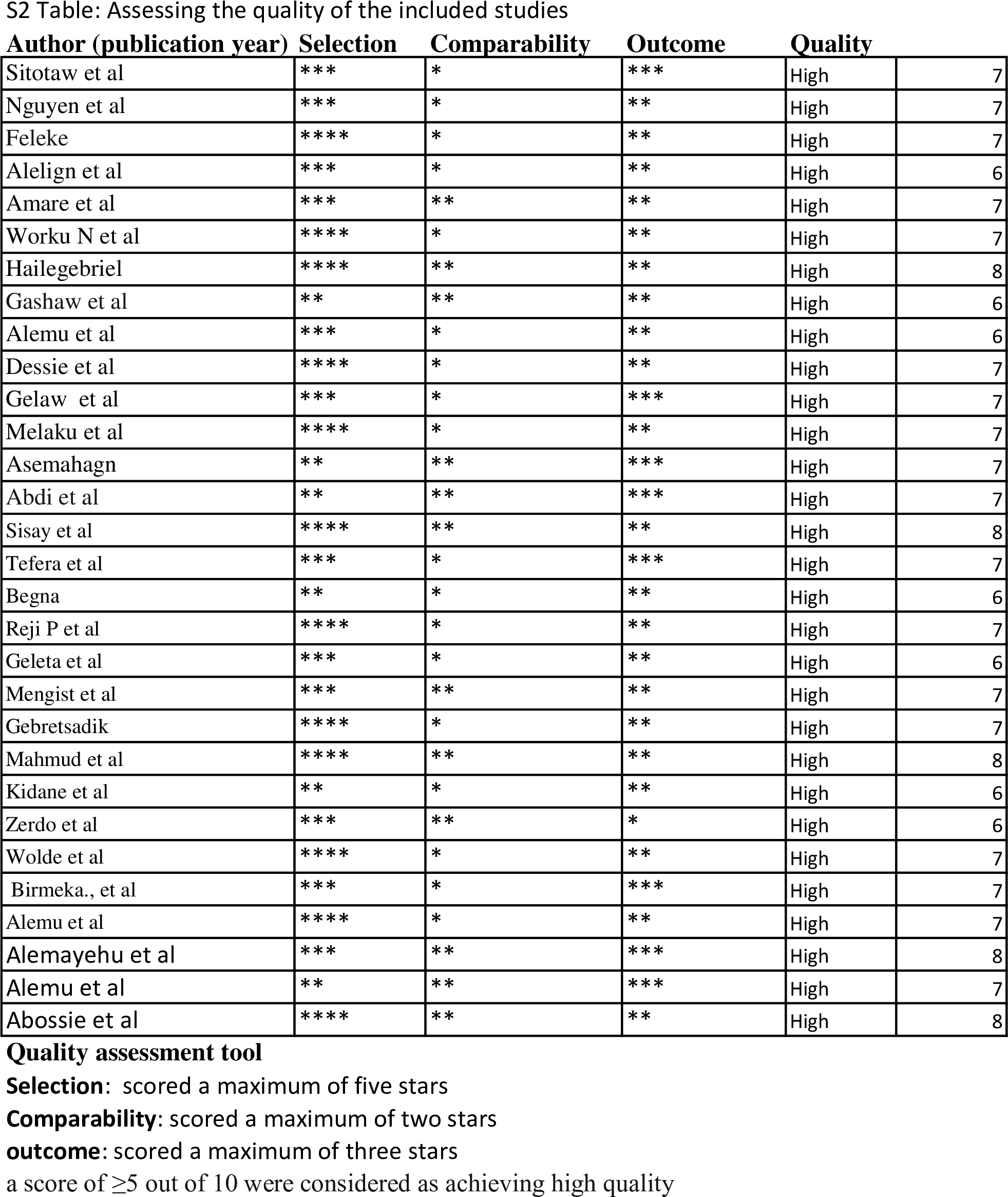

Supplement: S2 Table — (TIF) [file pntd.0009379.s002.tif]
